# Metal contamination – a global environmental issue: sources, implications & advances in mitigation

**DOI:** 10.1039/d4ra04639k

**Published:** 2025-02-11

**Authors:** Gabrijel Ondrasek, Jonti Shepherd, Santosha Rathod, Ramesh Dharavath, Muhammad Imtiaz Rashid, Martin Brtnicky, Muhammad Shafiq Shahid, Jelena Horvatinec, Zed Rengel

**Affiliations:** a Faculty of Agriculture, The University of Zagreb 10000 Zagreb Croatia gondrasek@agr.hr; b ICAR—Indian Institute of Rice Research Hyderabad 500030 India; c Department of Computer Science and Engineering, Indian Institute of Technology (ISM) Dhanbad 826004 Jharkhand India; d Center of Excellence in Environmental Studies, King Abdulaziz University 22252 Jeddah Saudi Arabia; e Department of Agrochemistry, Soil Science, Microbiology and Plant Nutrition, Faculty of AgriSciences, Mendel University in Brno 61300 Brno Czech Republic; f Department of Plant Sciences, College of Agricultural and Marine Sciences, Sultan Qaboos University Al-Khoud 123 Muscat Oman; g UWA School of Agriculture and Environment, The University of Western Australia Perth WA 6009 Australia; h Institute for Adriatic Crops and Karst Reclamation 21000 Split Croatia

## Abstract

Metal contamination (MC) is a growing environmental issue, with metals altering biotic and metabolic pathways and entering the human body through contaminated food, water and inhalation. With continued population growth and industrialisation, MC poses an exacerbating risk to human health and ecosystems. Metal contamination in the environment is expected to continue to increase, requiring effective remediation approaches and harmonised monitoring programmes to significantly reduce the impact on health and the environment. Bio-based methods, such as enhanced phytoextraction and chemical stabilisation, are being used worldwide to remediate contaminated sites. A systematic plant screening of potential metallophytes can identify the most effective candidates for phytoremediation. However, the detection and prediction of MC is complex, non-linear and chaotic, and it frequently overlaps with various other constraints. Rapidly evolving artificial intelligence (AI) algorithms offer promising tools for the detection, growth and activity modelling and management of metallophytes, helping to fill knowledge gaps related to complex metal-environment interactions in different scenarios. By integrating AI with advanced sensor technologies and field-based trials, future research could revolutionize remediation strategies. This interdisciplinary approach holds immense potential in mitigating the detrimental impacts of metal contamination efficiently and sustainably.

## Abstract 2

Sources of metal contamination, metal transfer pathways, and remediation approaches.

## Graphical abstract

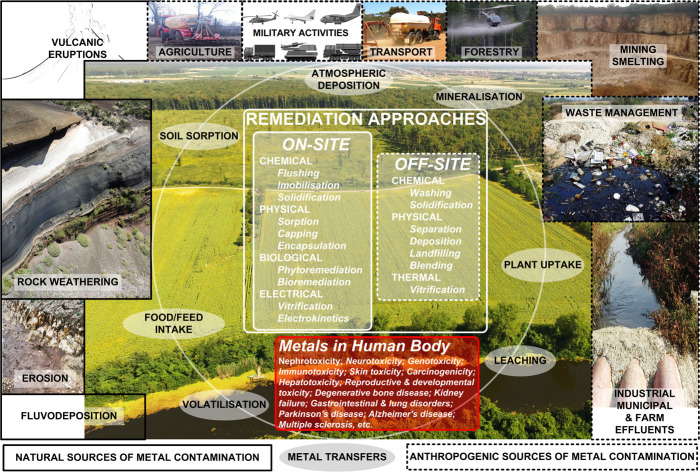

## 1. Introduction

The persistent and toxic nature of metals represents a significant threat to human health, the environment, and food production.^1,2^ In general, most toxic metals found in soil are adsorbed onto soil particles (with kinetics being rapid initially, followed by a slower phase), and then are (re)distributed into diverse chemical forms with different mobility, toxicity and bioavailability.^2^ The mobility of metals in soils, and their transfer through other environmental niches, including potential entry into the food chain, is significantly influenced by their chemical speciation.^3,4^ The distribution of metals in soil is controlled by a multitude of biogeochemical reactions and processes in the pedosphere, such as complex formation in the soil solution, ion exchange, adsorption/desorption, uptake by soil biota and their dissolution/precipitation.^5,6^ It was confirmed that high concentrations of metals in biological systems affect enzymatic processes and cell organelles and their components, including the nucleus, mitochondria, cell membrane, lysosomes, and endoplasmic reticulum, leading to DNA damage, changes in the cell cycle, carcinogenesis, and cell apoptosis.^7^ The high toxicity and carcinogenicity of arsenic (As) and metals such as lead (Pb), cadmium (Cd), mercury (Hg), chromium (Cr) are frequently the result of oxidative stress due to the formation of reactive oxygen species (ROS).^8^ These metal(loid)s are systemic toxins capable of damaging various organs even at low doses, prompting leading environmental and public health organizations to classify them as highly toxic, carcinogenic, and a significant threat to all living organisms.^9^

The chemical forms of metals in contaminated soils are influenced by many factors, primarily the soil organic matter (SOM) content and pH, and metal interactions with other soil variables.^10^ For example, the mobility of metals can be reduced through organic complexation by increasing the SOM in the soil. The use of soil bio-based conditioners such as biochar influences soil pH and increases SOM, which expands soil surface area (interface for metal adsorption), soil porosity, microbial activities and plant growth, ultimately leading to relatively rapid remediation of metal-contaminated soils.^11^ For instance, adsorption and desorption are the primary processes influencing soil metal accumulation, with studies typically indicating stronger accumulation of metal(loid)s in fine soil particles (clay, silty clay), although some studies have reported greater accumulation in coarse particles under specific conditions.^10,12^ Additionally, it was shown (albeit over a relatively narrow pH range) that the free cationic forms (most mobile and bioavailable) dominate in acidic (pH < 5) conditions, whereas the poorly mobile and bioavailable forms (*e.g.* carbonates, phosphates) or crystalline/amorphic forms (malachite, otavite, smithsonite) dominate in alkaline (pH > 8) environments.^13^ More specifically, a pH increases from 4 to 7 can decrease the amount of the most bioavailable Cd^2+^ by >60% at the expense of les bioavailable organo-complexed forms of Cd (that increased 7-fold). Consequently, the effective pH management of metal-contaminated acidic soils, using the addition of alkaline matrices can significantly reduce metal mobility and bioavailability, thereby mitigating their adverse impacts on (agro)ecosystems (more in Section 8).

Several pedovariables (pH, salinity) may contribute to high Cd transfer between soils and plants, as confirmed in radish,^14^ maize^13^ and strawberry^15^ due to formation of soluble and more mobile Cd-complexes. Moreover, this concept has practical applications in chemical remediation of metal-contaminated soils. Chloride salts such as CaCl_2_ and FeCl_3_ have proven to be effective soil-washing agents, reducing metal concentrations in soil and crops by promoting proton release and forming soluble Cd complexes^12^ (more in Section 7).

The extent of the toxicity of metals in the soil environment is determined by the chemical forms and the total concentration of the metals. It has been shown that metals of anthropogenic origin that accumulate in soils are more mobile and bioavailable than metals from lithogenic or pedogenic sources. Furthermore, simulation models show that anthropogenic (*vs.* natural) atmospheric emissions generate 3-to-7-fold greater quantities of toxic metals.^16^ However, it should be noted that the availability of metals is influenced by numerous abiotic biotic factors and interactions, such as temperature, adsorption, phase association, sequestration, solubility and complexation kinetics, genotype, plant species, ecotype, *etc.*^17^ In metal-polluted areas with more than 300 years of Pb mining and smelting, indoor environmental conditions can vary significantly. For example, attic dust primarily consists of calcium sulfates and metal-containing particles, whereas house dust is mainly composed of carbon-containing particles.^18^ Additionally, attic dust in these areas can have 7 times more metal-containing particles and 13 times more metal species of geogenic or anthropogenic origin compared to outdoor snow deposits. Consequently, in such metal-polluted regions, uniform mitigation approaches may prove ineffective on a small scale.

Here, we discuss the most important sources of metal contamination, their pathways within the biosphere, and the current remediation methods based on the recent scientific advancements. The objective is to disseminate awareness of the sustainable and efficient use of contemporary technologies, materials, and approaches in remediating metal-contaminated soils. This includes the integration of biorenewable technologies aligned with the medium-term green plans and policies. A critical discussion of specific sites facing significant challenges with the existing on-site or off-site remediation methods underscores gaps, limitations, and opportunities for improvement. Notably, we emphasize the need for (i) more stringent regulatory measures or comprehensive risk assessment protocols, and (ii) multidisciplinary approaches to effectively remediate metal contamination.

## 2. Soil metal contamination: a critical environmental threat

**Fig. 1:**
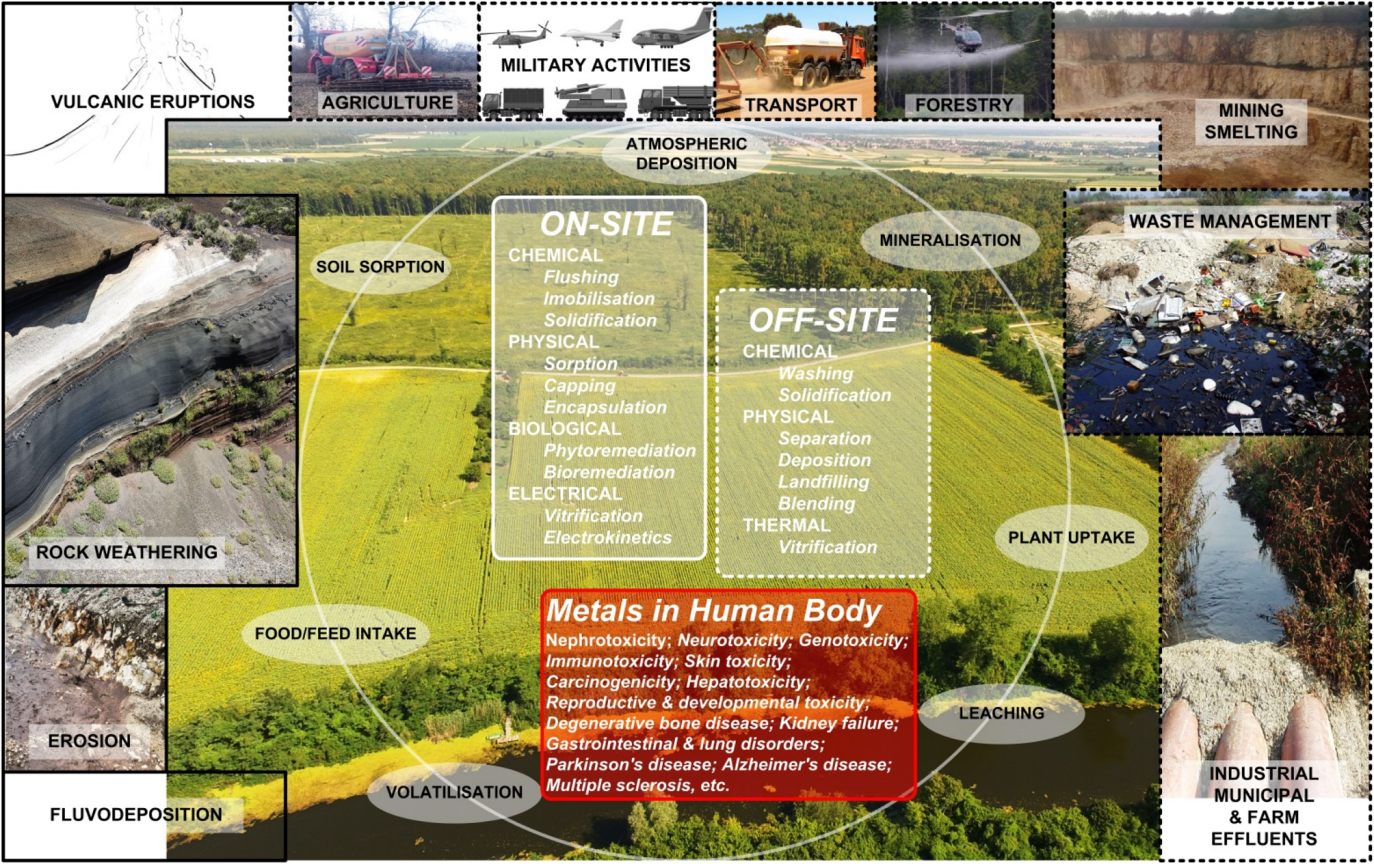
Schematic representation of the most important natural (rectangles bordered by solid black lines) and anthropogenic (rectangles with dashed black lines) sources of metal contamination, with metal transfer pathways (ovals) in the environment as well as into the human food chain, and on-site and off-site approaches to remediate metal-contaminated soils.

Metals enter the biosphere through a combination of natural and anthropogenic sources and processes (Fig. 1). Natural sources include weathering of parent rocks, volcanic activity, erosion, sediment resuspension, and metal corrosion, whereas agriculture emerges as the most prominent anthropogenic contributor to global metal emissions^7^ (Fig. 1). Since the industrial revolution in the 1760s, pollution of soils has been on the rise due to contamination by metal(loid) emissions from rapidly expanding industrial sources, such as manufacturing plants, coal burning, petrochemical releases/spills, atmospheric deposition, mining activities, waste disposal, application of wastewater for irrigation, agrochemicals such as pesticides and fertilizers, and soil amendments (Fig. 1). Zinc, Pb, Cd, As, and Cr are frequently found in contaminated sites,^19^ with Cu, Hg and Ni also commonly present.^20^ Based on the emission sources, two groups of metal(loid)s can be distinguished: (i) the As–Cr–Ni group primarily originates from natural processes/resources, whereas the Pb–Zn–Cu–Cd–Hg group is largely attributed to human activities^21^ (Fig. 1).

The most exploited ores globally are those vital for the construction, manufacturing, technology, and energy sectors, often containing Pb, Zn or Cd as associated metals or impurities, which complicates extraction, separation, purification and environmental management. For instance, in Celje area (Slovenia), the 100 years anthropogenic emissions from the former Zn smelting plant facility, with an estimated amount of >1700 t of Zn (∼0.3% of total Zn production) and >9 t of Cd, resulted in a heavily contaminated area with maximum concentrations of Zn (up to 5.6% w/w in attic dust and 0.85% w/w in the soil) and Cd (456 mg kg^−1^ in attic dust and 59 mg kg^−1^ in soil).^22^ Similarly, in the area around Kosovska Mitrovica (Kosovo) in the former mining area covering 302 km^2^, the maximum concentrations of heavy metals in the topsoil layer were also many times higher (*e.g.* in mg kg^−1^; Pb 35 000; Zn 12 000; Cu 1,600, Cd 47)^23^ than in uncontaminated soils.^9^ In some heavily polluted areas of China, the chronic daily intake of metals by residents near Zn–Pb mining sites has been documented to surpass the safe reference dose by as much as 15-fold.^24^

With increasing industrialisation, urbanisation, energy consumption and intensive agricultural production, especially in developing countries, metal pollution has accelerated worldwide, posing an increasing challenge to soil quality, food security and human health.^25^ The presence of Cd in critical concentrations in soil is associated with various harmful structural, physiological and chemical changes in plants. There are approaches to mitigate the toxic effects of Cd in plants, such as applying (in)organic amendments to reduce Cd mobility or using plants that can accumulate Cd from the soil without translocating it to edible parts.^14^

Zinc fertilization has been shown to decrease Cd uptake and oxidative stress while increasing the net photosynthetic rate,^26^ making it a potential candidate for mitigating the toxic effects of Cd. However, the competition between Zn and Cd uptake due to their similar chemical properties can be affected by the concentration of Zn, because high concentrations of Zn can become toxic to plants by increasing the formation of ROS and reducing growth, respiration, and photosynthesis.^27^ Therefore, further research is necessary to understand the role of Zn in phytoaccumulation of Cd, considering important factors (such as plant species, genotype, metal concentrations, and duration of exposure) to effectively mitigate Cd toxicity.^14^

In soil solution, a small proportion of Pb is phytoavailable because most of Pb forms various complexes with soil components.^28^ Although it is not a phytonutrient, Pb is taken up *via* the apoplastic pathway or Ca-permeable channels from the rhizosphere.^29^ The Pb dynamics in the soil and uptake by plants are influenced by soil pH, ion speciation, soil particle size, root surface area, and cation exchange capacity.^30^ When it enters the plant, it accumulates mainly in the root cells because it is blocked by Casparian strip in the root endodermis.^31^ Furthermore, it is retained by negative charges in the walls of the root cells.^32^ Damage to plant tissues and negative impacts on morphological, physiological and biochemical functions are the main problems resulting from the excessive Pb phytoaccumulation.^28^ The Pb accumulation induces phytotoxicity by altering the permeability of the plasma membrane. This alteration is attributed to the interaction between Pb and various active enzyme groups, particularly phosphates, which play a crucial role in plant metabolism.^33^ Lipid peroxidation and DNA damage due to excessive generation of ROS and inhibition of ATP production have been modelled in Pb toxicity.^34^ Lead damaged chlorophyll production, transpiration, protein content, seed germination, root elongation, and seedling development. Lead toxicity negatively affects plant growth by inhibiting Calvin cycle enzymes, leading to a deficiency of CO_2_ resulting in stomatal closure, reducing the uptake of essential macro- and micronutrients such as Mg and Fe, and hindering the electron transport system.^35^ However, there are adaptive mechanisms in plants involving a number of components that reduce the uptake of Pb into cells through cross-functional actions and provide resistance to Pb toxicity.^36^ In particular, Pb is sequestered in vacuoles through the formation of complexes with phytochelates, glutathione and amino acids.^37^ The activation of various antioxidants as a secondary type of defence mechanism serves to combat the Pb-induced increase in ROS production.^38^

As a result of modern activities such as mining, the use of agrochemicals and waste management, contamination with arsenic (As) is becoming a growing problem. Arsenic is found throughout the earth crust, and is a highly toxic metalloid. In water and soil matrices, it occurs mainly in forms such as arsenite (AsIII) and arsenate (AsV), which are more toxic than organic As species.^2^

After drinking water, the consumption of rice is the second most common way of As exposure.^2^ Arsenic contamination of rice agroecosystems is one of the greatest threats to safe and sustainable rice production. Due to the phytotoxicity of As, grain quality changes and yield decreases.^39^ Plants take up AsV mainly *via* phosphate transporters, whereas AsIII is taken up *via* aquaporins. Therefore, lower phytoaccumulation of As in rice grains and higher food safety can be achieved by genetically modifying rice varieties with altered expression and/or activity of specific transporters (the concept is explained in more detail in the following sections). The quality of water resources used for irrigation of rice agroecosystems has a significant impact on crop safety and consumption, as rice cultivation generally requires water. A study by ref. 2 has shown that more than 300 million people are affected by As contamination of groundwater sources and that, in Bangladesh alone, chronic As exposure is responsible for an annual death of up to 43 000 people.

A very common component of volcanic rocks and dust matrices is Cr (Fig. 1), which is used in leather tanning, the metal and alloy industry, ceramics and glass manufacturing.^40^ Chromium poses a significant threat to the environment by contaminating soil and water. Once Cr enters the food chain, it can pose a serious risk to human health. The oxidation states in which it occurs range from 0 to +6, with Cr(iii) and Cr(iv) being the most stable and toxic to humans, animals and plants. The bioaccumulation of Cr(iii) in living organisms and accumulation in the environment is exacerbated by the occurrence of (micro)plastic pollution, as polyethylene terephthalate and polystyrene serve as a vector for the transfer of pollutants to the aquatic environment.^41^ Due to its high toxicity, mutagenicity, genotoxicity and carcinogenicity^42^ the remediation of Cr is an important and urgent area of research in environmental science and engineering.

## 3. Agrochemicals as a source of metal contamination

Agriculture contributes significantly to soil metal inputs because many metal(loid)s are effective agrochemicals.^43^ Thus, in order to ensure optimal phytonutrition, soil pH balance, SOM content, and effective pest and weed management, the application of agrochemicals in intensive conventional farming systems must be carefully managed and monitored. The long-term annual application of different agrochemicals (fertilizers, soil amendments, plant protection agents, growth regulators) has been identified as a significant source of soil metal inputs/contamination^44,45^ (Fig. 1 and Table 1), even following the recommended dosage according to the specific agro-ecological conditions and the current national legislations.^46^ For instance,^46^ suggest for some of the agroecosystems in South Brazil that [excluding Fe and Mn due to their high soil background concentrations], the soil accumulation of metals through the application of agrochemicals increased in the following order: Hg > Pb > Co > Cd > As > Cr > Ni > Cu > Zn. Considering some related studies, the average annual input of metals through agrochemical applications ranges (in g ha^−1^): 0.03–0.71 for Hg, 0.8–12.8 for Pb, 0.6–3.7 for Co, 0.9–2.4 for Cd, 1–6.8 for As, 5.6–28 for Cr, 3.6–23.4 for Ni, 8–122 for Cu and 40–230 for Zn.^46,53,54^ Recently^21^ found that even at sites with the same current crop and agrochemical applications (herbicides and fertilizers), metal accumulation patterns differed, suggesting that the total amount and type of chemicals applied over time plays a significant role in determining Fe, Zn, Mn, Cu and Al content. The study also highlights the potential impact of crop rotation on soil metal accumulation, relating such outcome with well-known phytoremediation capacities of many crops^55^ (Fig. 1).

Metal concentrations in major agrochemicals, growth substrates, and livestock feed additives in nano-forms (based on ref. 46–52)

**Table:** 

Type	mg kg^−1^											
	As	Ba	Cd	Pb	Co	Cu	Cr	Fe	Mn	Hg	Ni	Zn
**Herbicides**												
Median	<3.9		<0.2	<2.7	<0.2	1.71	0.9	43.1	2.02	0.08	0.55	1.8
Minimum	<3.9		<0.2	<2.7	<0.2	1.32	0.75	8.29	1.45	<0.05	<0.2	0.87
Maximum	<3.9		<0.2	<2.7	<0.2	1.85	1.68	53	2.08	0.08	0.55	4.54

**Insecticides**												
Median	7.4		<0.2	3.7	0.91	3.86	2.66	31.1	5.93	0.11	3.46	6.88
Minimum	<3.9		<0.2	<2.7	<0.1	1.02	1.19	1.71	1.49	<0.025	<0.1	3.06
Maximum	7.4		<0.2	5.8	10.9	242	42	592	436	0.21	10.8	44.5

**Fungicides**												
Median	<3.9		2.95	22.3	77.1	10.3	11.2	417	11.3	0.17	12.2	5.86
Minimum	<3.9		<0.2	9.2	<0.2	7.4	5.47	188	1	<0.05	<0.2	1.52
Maximum	<3.9		3.2	367	159	307	50.5	529	92.6	0.73	84.1	11.3

**Anti-sprouting agents**												
Median	<3.9		<0.2	<2.7	<0.2	3.34	1.49	27.7	2.01	<0.05	0.95	5.67
Minimum	<3.9		<0.2	<2.7	<0.2	2.35	1.33	18.5	1.47	<0.05	<0.2	2.43
Maximum	<3.9		<0.2	<2.7	<0.2	3.9	1.52	80.1	98.6	<0.05	0.95	5.95

**Phosphatic fertilizers**												
Median			13	13		26	60				22	236
Minimum	2	200	0.1	7	1	1	66		40	0.01	7	50
Maximum	1200		170	225	12	300	600		2000	1.2	38	1450

**Nitrogen fertilizers**												
Median			0.9	1.9		2	3.4				6	5
Minimum	1		0.05	2	5	1	3			0.03	7	1
Maximum	120		8.5	1450	12	15	19			3	38	42

**Lime fertilizers**												
Median			0.2	8.2		5.6	6.5				6.3	22
Minimum	0.1	120	0.04	20	0.4	2	10		40	0.05	10	10
Maximum	24	250	0.1	1250	3	125	15		1200		20	450

**Manures**												
Minimum	3	270	0.3		0.3	2	5.25			0.09		15
Maximum	150		0.8		24	60	55			26		250

**Growing substrates**												
Median	10.7		<0.2	<2.7	15.7	13	11.4	4.3	159	0.2	3.1	35.2
Minimum	<3.9		<0.2	<2.7	<0.22	10.9	5.8	1.4	70.3	<0.05	2.2	29.6
Maximum	11.2		<0.2	12.3	31.7	76.6	544	6.1	220	0.4	517	69.1

a Broiler chickens.

b Piglets.

**Table:** 

Ameliorative impact of nano-mineral additives on livestock growth performance or productivity		
Se	Cu	Zn
0.1–1.2^a^	50^a^	100–200^a^
		500–3000^b^

**Fig. 2:**
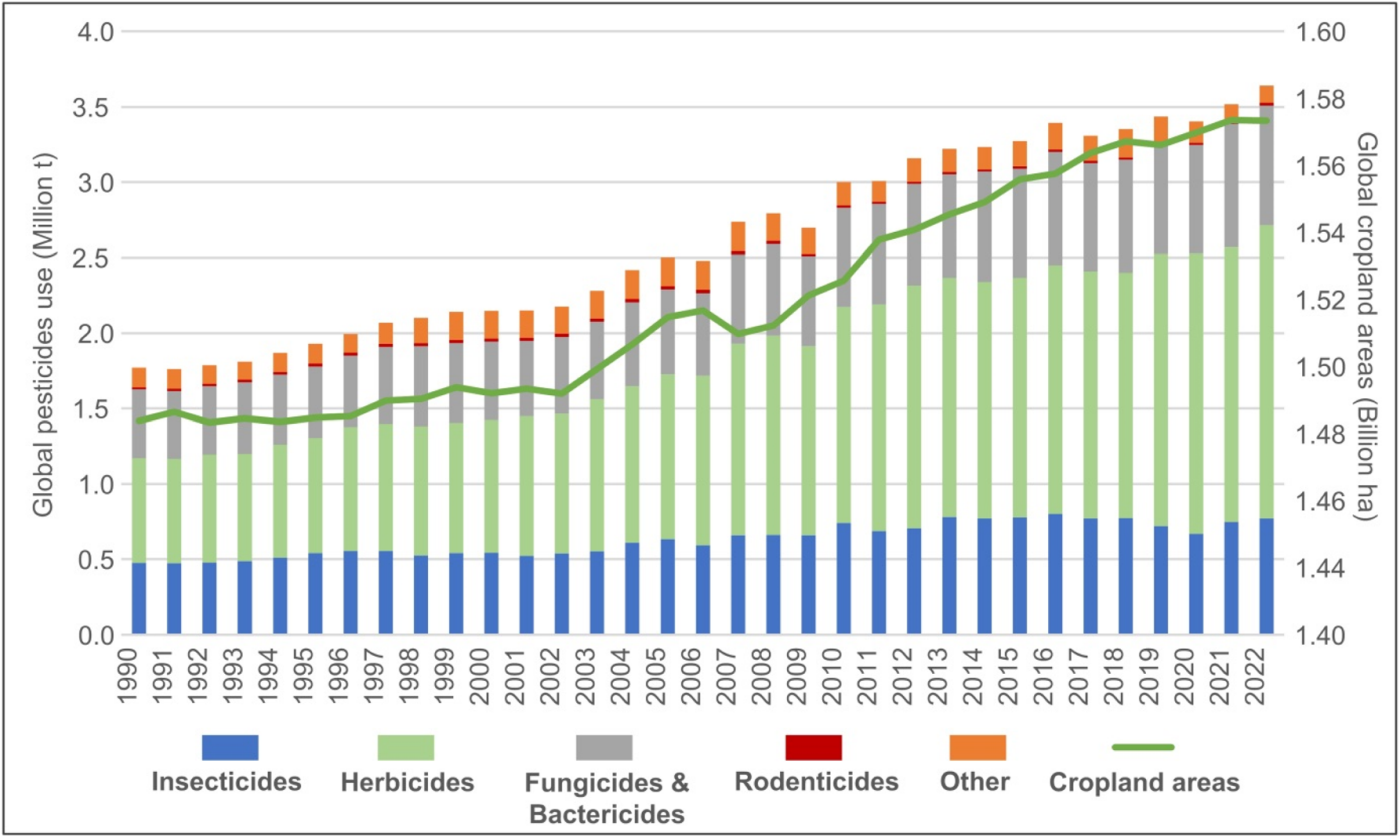
Global distribution of pesticide use on croplands globally from 1990 to 2022 (based on data from ref. 56 and 57)

The regulations regarding the use of different agrochemicals vary significantly among countries. The maximum permitted limits of metal(loid)s in fertilizers differ by several orders of magnitude for the same metal(loid), *e.g.*, in Brazil, the limit for As is 10 mg kg^−1^, but in Canada it is 775 mg kg^−1^; for Cd, Brazil allows up to 20 mg kg^−1^, whereas Canada permits 207 mg kg^−1^; for Pb, the limit is 100 mg kg^−1^ in Brazil compared to 5169 mg kg^−1^ in Canada.^46^ According to the same authors, differences among maximum permitted limits of metal(loid)s in the growing (in)organic substrates are even higher. This inconsistency likely reflects the varying metal concentrations in raw materials (ores, rocks), limiting national exploitation options.^43^ In addition,^45^ many countries have imposed restrictions or prohibitions on a number of common pesticides that are used intensively in agri-/forest sectors, and are the source of significant concentrations of metals (Fig. 2 and Table 1). Pesticides are extensively used in conventional agriculture as an effective and economical approach to ensure stable crop yields, thus ensuring food security (Fig. 2 and ref. 58). Global cropland areas have expanded by 6% over the period from 1990 (1.48 billion ha) to 2022 (1.57 billion ha) (Fig. 2). Global agricultural pesticide use reached 3.63 Mt of active ingredients in 2022, with herbicides accounting for 55%, insecticides for 22%, fungicides and bactericides for 22%, and other categories for 3.6% of that amount (Fig. 2). This marked a 4% increase compared to 2021, a 13% rise over the past decade, and a doubling since 1990 (Fig. 2 and ref. 59). Between 1990 and 2022, the intensity of pesticide use grew at varying rates: application per unit cropland area surged by 94%, use per unit of agricultural production value rose by 5%, and use per capita increased by 35%.^56^ However, pesticide use in Europe declined by 5% since 1990, with a 7% reduction in the last decade, largely attributed to stricter regulations under the European Common Agricultural Policy^60^ which enforces rigorous pesticide monitoring programs and control. In contrast, the Americas have been the leading pesticide consumer since the mid-1990s, recording a 210% increase in usage between 1990 and 2022, with a notable 31% rise only in the last decade^56^ (Fig. 2).

While pesticides containing Hg, As, Cu and Pb, once widely used, have now been banned in many countries, the presence of other metal(loid)s as impurities in these products may still pose a significant environmental risk, depending on the management practices adopted and the specific products used.^46^ An example of this is Bordeaux mixture, a CuSO_4_-based pesticide that has been banned in most EU countries and the UK due to excessive Cu concentrations in the environment.^16^ Application of foliar spraying with a Bordeaux mixture for plant protection^17^ would result in >64% of vineyards older than 40 years in NW Croatia having received between 80 and 200 kg Cu per ha, with most of the applied Cu being in the surface soil layer where decomposition of biomass occurred (Fig. 1).

The activities in the transport sector and forestry are still, or were until recently (*e.g.* tetraethyl lead), responsible for a significant emission of metals that could enter water bodies and other ecological environments through (sub)surface runoff and/or leaching (Fig. 1). In the UK, approximately 5% of currently authorised insecticides and fungicides are based on compounds containing metals such as Pb, Zn, Cu, Hg and Mn.^61^

## 4. Bio-based amendments as a source of metal contamination

**Fig. 3:**
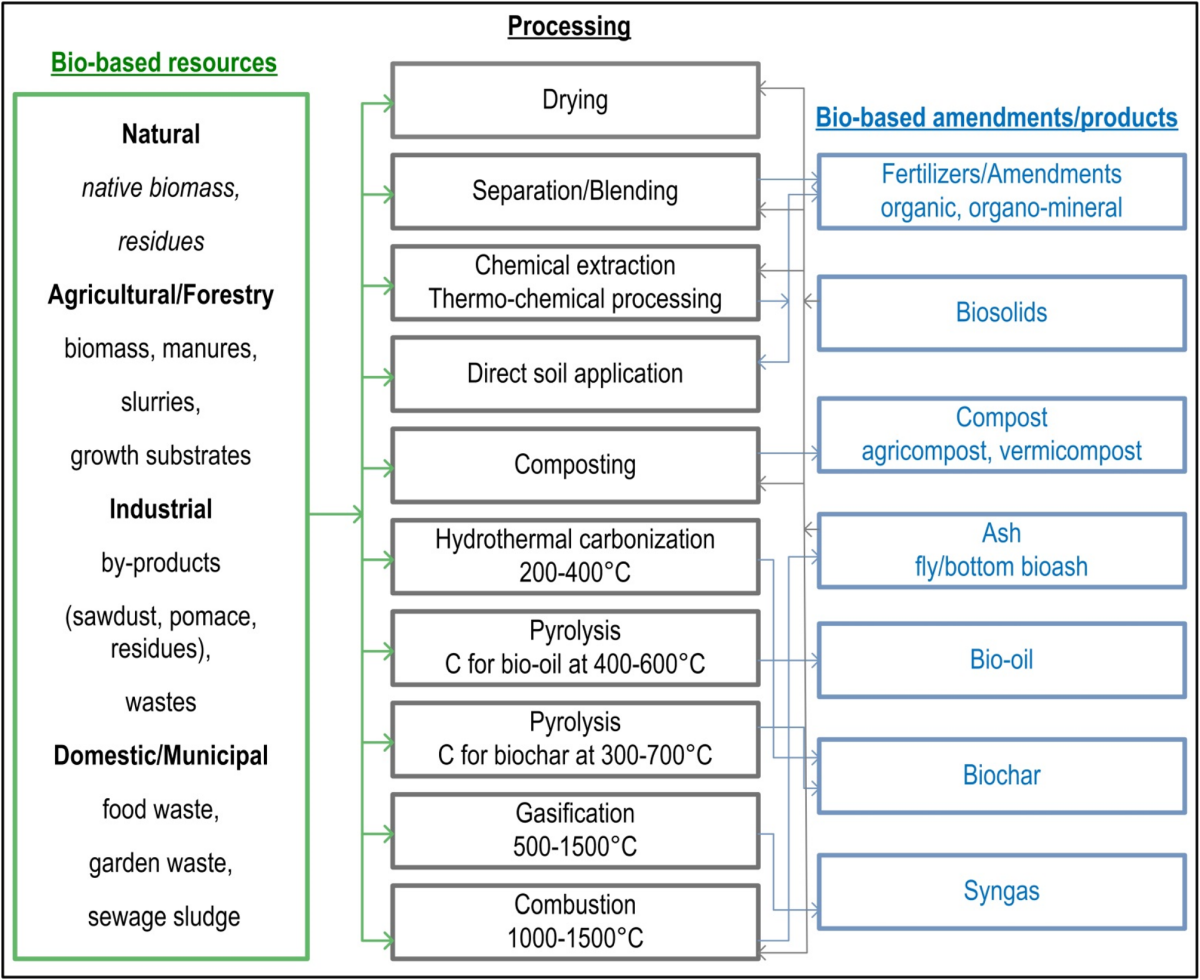
Transformation of bio-based resources into bio-based amendments (reproduced (adapted) with permission from ref. 62 Copyright© 2024, Royal Society of Chemistry).

Various types of biosolids, including compost, animal manure, and municipal sewage sludge, are persistent sources of metal contaminants such as Cd, Zn, Pb, Hg, Cr, Ni, Cu, Mo, Se, Tl, and Sb.^61^ Unlike fertilizers and pesticides, with targeted and direct applications, biosolids continually contribute to metal contamination despite careful consideration of their use^61^ (Fig. 3). Due to the favourable biochemical composition, the application of animal wastes such as cattle, pig, and poultry manure in the form of solids or slurry is encouraged on croplands, pastures and urban public areas. Indeed, the increased concentration of metals in most fertilisers and wastewater from livestock farms comes from feed additives (growth promoters) added to animal feed for their regulatory, structural and catalytic role^63^ in maintaining animal health and productivity.^64^ To prevent excessive growth of pathogenic microorganisms and the occurrence of diarrhoea and to have a positive effect on production parameters (yield and feed conversion), it is a common practice to enrich the feed with Zn and Cu in excess of the nutritional requirements, which, especially after the ban on antibiotics as growth promoters,^65^ has proven successful in practice, particularly in pigs.^66^ In addition, mineral additives are widely used in animal feeds to supply essential nutrients like Cu, Zn, Fe, Cr, Mn, and Co, improving livestock growth and performance;^47^ however, the low purity of these additives often introduces non-essential and toxic metal(loid)s (Cd, Hg, As, Pb) into the food chain.^24^ The risk to the (agricultural) environment lies in the increased excretion of faeces from the livestock and the accumulation of metals in the manures.^63^ For these reasons, the use of some metal-based additives in livestock feed is limited (*e.g.* for Zn to 150 mg kg^−1^ and for Cu to 170 mg kg^−1^ for pigs up to 12 weeks^65^) but these prescribed doses still exceed the nutritional requirements of pigs (50–100 mg per kg Zn and 3–20 mg per kg Cu).^67^ An additional potential source of (agricultural) pollution comes from the liquid component of manure containing significant amounts of metals being used in fertilisation/fertigation.

From the municipal sector, wastewater treatment plant facilities generate sewage sludges (commonly called biosolids) that contain solid organo-mineral compounds^68^ dependent on the wastewater source and the level of purification treatment.^69^ Organic N and inorganic P and K represent the majority of the total nutrient content in biosolids, with approximately 4.7% total N, 2.3% total P and 0.3% total K (on a dry weight basis),^70^ suggesting this matrix is a valuable source of phyto-nutrients.

As of 2025, global biosolids generation (on a wet weight basis) is estimated at approximately 150–200 Mt, reflecting a significant increase from the previous estimate of 100–125 Mt.^71^ It is a common practice to utilize biosolids for land applications as a way of beneficial recycling;^72^ however, the legislative framework for the land application of biosolids is not uniform (inter)nationally. At the EU level, the regulation of heavy metals in sewage sludge for agricultural use has been in place for nearly 40 years, following the adoption of the Council Directive.^73^ The Directive was established to safeguard biota, agricultural soils, and the broader environment by limiting the potential negative impacts of biosolids application due to elevated concentrations of heavy metals. However, the scope of the directive is limited because it only sets permissible levels with relatively broad concentration ranges for only a few toxic metals (Table 2). Despite this regulatory effort, the framework does not account for emerging pollutants, variability in soil conditions, or the evolving understanding of long-term ecological impacts. Furthermore, inconsistencies in the national implementation of the directive result in varying levels of environmental protection, both across EU member states and globally.

**Table 2:** Limit values for metals in (*A*) sewage sludge used in agriculture, (*B*) agricultural soil treated with sludge, and (*C*) that can be annually added to agricultural soil treated with sludge^73^

Metal	*A* mg per kg dw	*B* mg per kg dw of soil with a pH 6–7	*C* kg per ha per year
Cd	20–40	1–3	0.15
Cu	1000–1750	50–140	12
Ni	300–400	30–75	3
Pb	750–1200	50–300	15
Zn	2500–4000	150–300	30
Hg	16–25	1–1.5	0.1

For example, in the USA more than 50% of approximately 9.6 Mt of dry biosolids produced annually are applied on agricultural land,^74^ and similarly in the EU more than 42% of biosolids are utilized as fertilizers/amendments in agriculture.^75^ In addition, certain European countries (Norway, Sweden, Denmark) have not only prohibited the disposal of biosolids in landfills, but also implemented taxes on landfilling and waste incineration;^71^ by contrast, other EU member states like Croatia have strictly forbidden any application of biosolids on agricultural land for food production.^76^ Such restrictions on use of sewage sludge is related to the presence of heavy metals (Table 2), and emergent contaminants (pharmaceuticals, drugs, micro/nano plastics); hence, additional treatments (*e.g.* drying, alkalinisation, composting, blending) are necessary prior to safe application as soil amendments (Fig. 3).

The recent trend of mixing composted sewage sludge with various organic materials such as food waste from urban areas, straw, sawdust, garden and municipal waste, and plant biomass from agriculture and forestry (Fig. 3) has led to the spread of metal contamination (Fig. 1 and ref. 77). Metals from sewage sludge (Cd, Pb, Cr, Zn, Ni, Cu) can be found in high concentrations in mixed composts,^78^ depending on the processes used in the treatment of sewage sludge.^77^ The application of sewage sludge to soil can result in metals leaching through the soil profile and into groundwater. Recent studies, particularly from developed countries, are focusing on methods to safely apply composted sewage sludge to soil without risking groundwater contamination,^79^ with this issue reported in soils treated with sewage sludge in New Zealand.^77^ Factors that influence soil contamination with metals include the feedstock materials used in compost production, soil depth and profile properties, temperature, moisture content, and the surrounding landscape.^79^

## 5. Wastewaters as a source of metal contamination

The use of various wastewaters is often associated with high risks of metal toxicity^42^ and/or salinity.^80^ Nevertheless, wastewaters can partially or fully meet the water and nutrient requirements of crops.^81^ It is estimated that 20–25 million hectares of agricultural land worldwide are irrigated with wastewater.^79^ In Asia and Africa, farmers are generally more focused on maximizing vegetable yield and therefore profits, rather than environmental protection.^16^ Studies indicate that agricultural irrigation with wastewater accounts for approximately 50% of all vegetables supplied to urban markets.^82^

In developed countries, strict regulations result in relatively low concentrations of metals in processed wastewater compared to untreated wastewater.^83^ However, an analysis of heavy metal content in wastewater discharged across regions in China during 2011 indicated a total discharge volume of ∼66 billion tons, containing substantial quantities of hazardous metals and As, including Pb (155 t), Hg (2.8 t), Cd (36 t), Cr(vi) (106 t), Cr (293 t), and As (146 t).^84^ These findings underscore the severe environmental challenges posed by industrial and municipal wastewater discharges in regions with less efficient and/or stringent wastewater treatment and management systems. Elevated metal concentrations in wastewaters pose a serious environmental challenge, particularly when wastewater is reused for agricultural irrigation. Over time, the application of metal-enriched wastewater can result in the accumulation of these metals in soil and their transfer to crops, thus highlighting potential risks to soil quality, food safety/security, and ecological health. Unfortunately, the primary source of metal accumulation in food^1^ is the widespread use of untreated (unfiltered, unsterilized) wastewater for irrigation in (peri)urban and industrial areas adjacent to arable land.^85^ Furthermore, hazardous metals discharged from the pulp and paper industries contaminate large areas of agricultural and freshwater environments, affecting aquatic biota.^86^ In addition, nearly 80% of tanneries are involved in the Cr tanning, emitting up to 3200 tonnes of Cr per year to the environment.^42^

Activities related to mining have caused the most widespread metal contamination in soil, especially in recent decades (Fig. 1). Since the late 1990s, rapid industrialization and urbanisation have driven significant growth in global metal consumption rates, with annual increases of 6% for Mn, 5% for Al, Cr and Ni, 4% for Zn, and 3% for Cu and steel.^87^ Moreover, it is expected that a transition from fossil-based to renewable energy sources will significantly increase the demand for specific metals because C-neutral energy infrastructures require substantially more raw materials per megawatt of installed capacity compared to traditional fossil fuel-based facilities.^87,88^ Additionally, energy transition, increasing urbanization, and the high demand for ores and minerals in industry suggest that more metals will be mined by the middle of this century than in the entire previous century. Accordingly, transitioning to renewable energy as the sole energy source is expected to require approximately 330 Mt of Cu (a nearly 20-fold increase over current global annual production), 8 Mt of Li (a 190-fold increase), 66 Mt of Ni (a 30-fold increase), and 31 kt Pt (a 15-fold increase).^88^ Mine tailings, consisting of heavier and larger particles that settle at the bottom of flotation cells during mining, are often discharged directly into natural depressions in the landscape or accumulated in tailings dams.^16^ The rapid extraction of ores and subsequent smelting processes have led to global soil pollution, posing significant risks to human health and the environment^89^ (Fig. 1).

## 6. Airborne sources of metal contamination

Most metals emitted into the atmosphere are released as particles in the gas stream.^90^ Fugitive emissions, which consist of gases or vapours, are released from stacks, ducts, chemical storage facilities, or waste dumps containing soils contaminated with various (frequently non-characterized) substances that cause toxic events, thereby becoming sources of airborne metals^90^ (Fig. 1). During high-temperature processing, some metals and metalloids (such as Pb, Cd, and As^91^) can vaporize and condense into fine dust particles if a reducing atmosphere is not maintained.^90^ After certain time, dust particles in the atmosphere settle on land and water, and gaseous metal elements can dissolve on these surfaces, collectively increasing environmental metal pollution.^84^ In general, emissions from stacks are dispersed over large areas by natural air currents but can be removed from the atmosphere by precipitation (rain, snow), thus polluting aquatic and terrestrial ecosystems (Fig. 1). In contrast, fugitive emissions are distributed over smaller areas and are released close to the ground.^92^ Notably, fugitive emissions resulting from incomplete combustion were high, indicating that total emissions from combustion are considerably underestimated if leakage is not accounted for.^93^ In both types of emissions, different metals are emitted from different sources. The reasons for this are varied and range from what is produced or destroyed to the types of filters that may be used to capture the emissions.^4^

Since the industrial revolution widespread metal pollution has been triggered by fossil fuels, and high concentrations of Pb, Cd and Zn have been measured in plants, soils and waters near smelters.^94^ Extremely high Pb concentrations in soils in urban areas and along major roads were previously associated with the combustion of petrol containing tetraethyl Pb.^90^ However, the use of tetraethyl Pb has steadily decreased over the last 30 years; as a result, a major problem in soils near major roads presently s that they contain high concentrations of Cd and Zn used in the production of tyres and lubricating oils.^95^

The legacy of metal pollution since industrial revolution extends into modern challenges posed by rapid industrial growth and urbanization. For instance, in China, waste gas emissions increased from 2003 (199 trillion m^3^) to 2010 (519 trillion m^3^), with an average annual emission of 359 billion m^3^.^84^ This increase has contributed to a large increase in the release of particulate matter (PM), which is a significant carrier of atmospheric metals. Recent findings reveal that PM10 (≤10 µm in diameter), primarily originating from the Earth crust, road traffic, and fuel combustion, poses significant risks in both developed and developing countries, with a more severe impact in Asian nations (notably China and India) compared to Europe and the USA, where levels have declined over the past two decades.^96^ Recent analyses of 118 full-scale industrial plant facilities revealed that the majority (∼98%) of particulate matter (PM) had diameters <2.5 µm, with 79% having diameters below 1 µm;^97^ it should be borne in mind that PM < 10 µm poses the greatest health problems. According to the same source, annual atmospheric releases of Fe, heavy metals (Cd, Cr, Cu, Ni, Pb, Zn), As and five crystalline metallic compounds (ZnO, PbSO_4_, Mn_3_O_4_, Fe_3_O_4_, Fe_2_O_3_) contained in fine PM from these industrial activities are estimated globally to be 51 Mt, 70 Mt, and 78 Mt, respectively.

The persistence of airborne metal pollution highlights the ongoing environmental and health challenges posed by industrial emissions and urbanization, particularly in rapidly developing regions. Mitigating the significant risks associated with airborne fine particulate matter and heavy metals requires urgent attention not only to control emission and achieve sustainable industrial practices but also to refine strategies addressing global climate change. Meteorological conditions, such as storms, wind speed, air temperature, and relative humidity, have been shown to significantly influence the spatiotemporal variability in concentration of various particulate matter sizes, both locally and regionally.^96^

## 7. Mechanisms of metal uptake and redistribution in plants

**Fig. 4:**
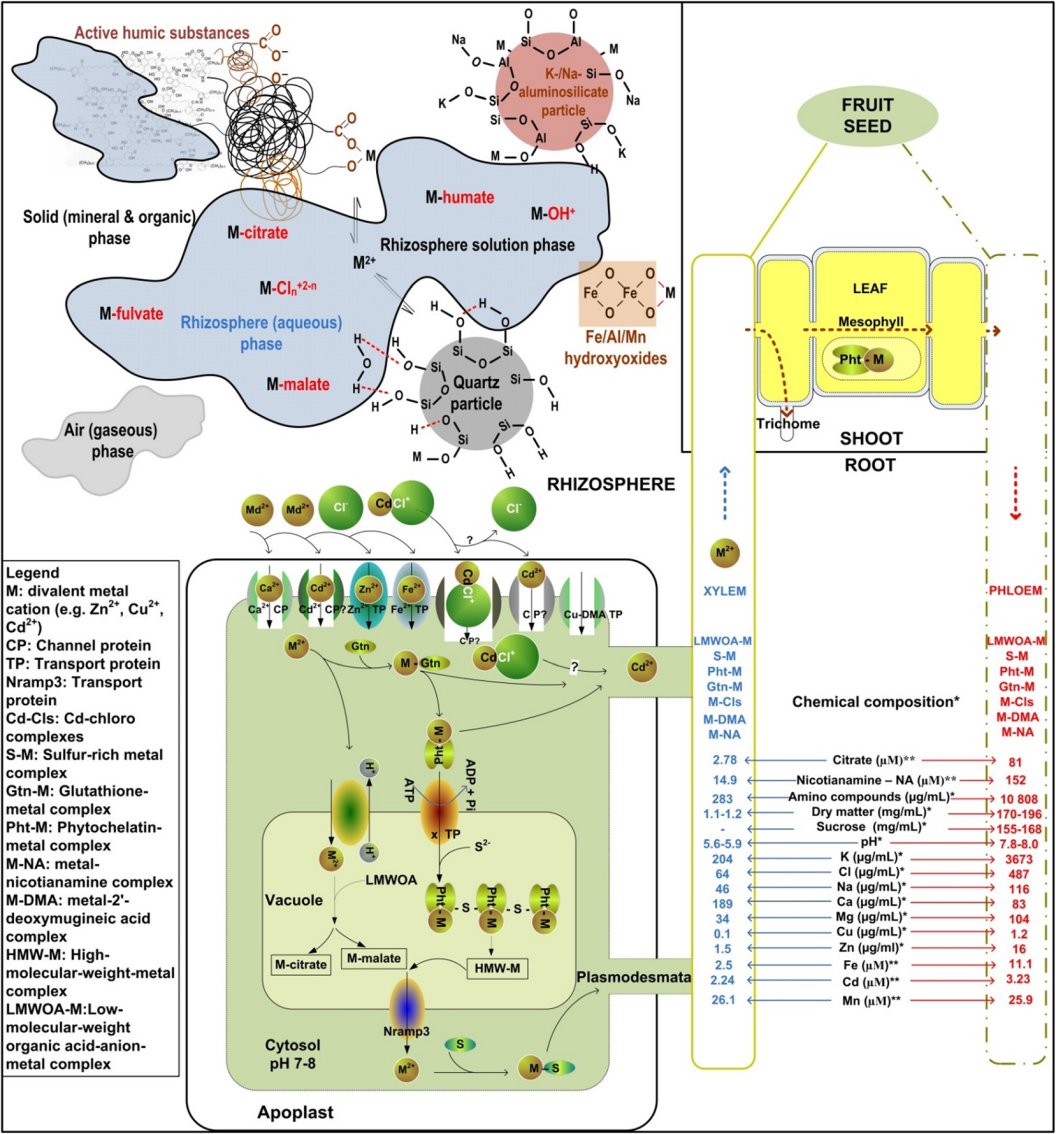
Schematic presentation of biogeochemical reactions in the rhizosphere and plant vascular tissues that impacts metals mobility, uptake and deposition in plants. Based on the chemical composition of xylem and phloem sap in wild tobacco (*Nicotiana glauca* Grah.)^98^*, and castor bean (*Ricinus communis* L.) at 10 µM Cd treatment^99^**.

One of the main pedovariables driving the solubility and bioavailability of metals in the rhizosphere is pH reaction (Fig. 4).^100^ Metals such as Zn, Cd, and Cu are among the most soluble and phytoavailable in the rhizosphere and exhibit relatively low selectivity for phyto-uptake. This trait is not limited to metallophyte plant species (more in next section), but is common to most cultivated plants, allowing these metals to relatively easily overcome numerous rhizosphere-plant barriers (Fig. 4). For instance, by using the high-resolution secondary ion mass spectrometry (nano-SIMS) as one the most advanced *in situ* approaches for metal mapping, it was documented that Cd and Zn, even after short-term exposure (24 hours) to very low equimolar concentration (2.2 µM) can rapidly cross root^101^ and shoot barriers and reach edible parts of widely consumed vegetable.^14^

The consummation of crops produced in metal-contaminated surroundings represents the main route of metals entering food and feed sources (Fig. 1). In general, concentration as well as distribution of most metals decrease following the reach of the upstream transpiration stream: roots > shoots > fruits > seeds^102^ (Fig. 3). Plants absorb metals from the rhizosphere through their roots, store a portion in the underground tissues, and translocate the rest to the shoots *via* transpiration stream (xylem sap), followed by redistribution *via* phloem sap and sequestration in different tissue and cell compartments^99^ (Fig. 4).

Some toxic metals resemble essential metals in physical (*e.g.*, ionic radii: Cd^2+^ 97 pm, Ca^2+^ 99 pm, Mn^2+^ 80 pm, Fe^2+^ and Zn^2^^+^ 74 pm) and chemical properties (*e.g.*, redox activity, Lewis acidity), thereby likely entering the pathways specific to these essential elements.^80,99^ Metal uptake across the plasma membrane of root cells has been shown to occur *via* concentration-dependent mechanisms that exhibit saturable kinetics,^103,104^ with confirmed Cd–Zn; Cd–Mn and Cd–Cu competition^103,105^ or Fe–Cd complementarity.^106^ In addition, the mobility and uptake of metals are highly dependent on: (i) the pH reaction of the xylem and phloem sap, and (ii) the presence of (in)organic ligands (*e.g.*, organic acid anions, chlorides, carbonates, sulfates) with a strong potential for creating metallo-complexes^80,99^ (Fig. 4). Metal complexation with ligands can enhance metal desorption from the solid interfaces (aluminosilicates, hydroxyoxides, humics) in the rhizosphere, and impact the uptake and long-distance transport of some metals in plants (Fig. 4). For instance, the cationic metal forms may adsorb onto negatively charged cell walls and plasma membrane and can easily precipitate in alkaline media (pH > 8; Fig. 4) as observed in the phloem sap of rice.^99^

Different biomolecules, channel protein (CP) and transport proteins (TP) incorporated in the plant membranes (*e.g.* Cu-DMA TPs, Zn–Fe permease, ATPases, cation diffusion facilitators, cation exchangers, *etc.*) embedded in the plant cell plasma membranes play crucial roles in the uptake and redistribution of metals^107,108^ (Fig. 4). For instance, uptake of metals from the rhizosphere is possible by specific transporters (for nutrients) (Ca-CP; Zn-TP; Fe-TP; Cu-DMA-TP), whereas toxic metals enter by competing for nutrient transporters^109,110^ (Fig. 4).

Metal-binding proteins (*e.g.* Cu chaperone ATX1-like proteins, glutathione – Gtn, metallothioneins – Mts, phytochelatins – Pht), organic acid anions, amino acids (*e.g.* histidine, cysteine, glutamine, asparagine), and peptides are essential for binding, sequestering, and detoxifying toxic metals in plant tissues^42,108,111^ (Fig. 4). These activities primarily occur in the cuticle, epidermis, and trichomes, areas where the cellular damage poses a relatively minor risk to plant survival.^107,112^ For instance, in the root cytoplasm metals are likely to be complexed by Gtn, *i.e.* a precursor of metal-Pht complexation (Fig. 4). Both Gtn and Pht are low-molecular-weight cysteine-based peptides that keep metal cationic species in the cytoplasm at low concentrations.^113^ As a Pht–Cd complex, Cd can cross the tonoplast *via* certain TPs and create Pht-based Cd complexes in the vacuole.^103,104^ In addition, free cytosolic metal forms can be anti-ported into the vacuole^103^ and sequestered with organic acid anions; these metal from the vacuolar pool may be remobilised into the cytosol (*e.g.* by Nramp3 proteins) and chelated with sulfur-containing ligands (Fig. 4).

Due to diverse organo-mineral composition and acidic reaction in the xylem sap, it is likely that metals are complexed (Fig. 4), *e.g.* Fe-citrates and Fe-phytosiderophores in some Poaceae (maize, rice, barley) and non-graminaceous plants (tomato, soybean, castor bean).^114,115^ The examples are Ni–histidine complex in the xylem sap of Ni accumulator *Alyssum lebiacum*^116^ and Cu complexed with 2′-deoxymugineic acid in the xylem sap of rice.^108^ It is shown that metal deposition in some plants is specifically targeted to particular leaf cells, such as trichomes (*i.e.* leaf hair or gland cells derived from a specialized epidermal layer on the leaf or stem surfaces) (Fig. 4). In the phloem sap that is alkaline, the metal forms are present mostly as organo-metallo-complexes, *e.g.* Cu–nicotianamine, Cu–histidine, and other Cu complexes (>3 kDa in size) in the rice phloem sap,^108^ although the existence of inorganic complexes should not be disregarded (Fig. 4).

## 8. Remediation of metal-contaminated soils

Numerous remediation methods are currently available for metal-contaminated environments and can be broadly categorized into on-site and off-site approaches (Fig. 1). These include a variety of physical (*e.g.*, soil washing, excavation, solidification), chemical (*e.g.*, flushing, immobilisation), biological (*e.g.*, phytoremediation, bioremediation), electrical (*e.g.*, electrokinetics), and thermal (*e.g.*, vitrification) processes, tailored to target specific contaminants and site conditions (Fig. 1). We focus on three promising approaches: phytoremediation and the use of bio-based materials such as bioashes and biochars for chemical conditioning of contaminated sites. These methods offer sustainable, cost-effective solutions while minimizing secondary environmental impacts. Phytoremediation leverages the capacity of plants to extract, stabilize, and/or degrade contaminants, making it an eco-friendly and low-cost remediation technique. Similarly, biochars (derived from organic materials through pyrolysis) and bioashes (produced by the oxidation of organic residues) demonstrate significant potential for immobilizing heavy metals and enhancing soil health.

### 8.1. Phytoremediation of metal-contaminated soils

Plant tolerance to metals is a crucial requirement for metal accumulation and phytoremediation, and it is regulated by various biomolecules (Fig. 4,^99,107^).The (hyper)accumulating plants (metallophytes) are used to extract or ‘excavate’ potentially toxic metals from contaminated soils.^117^ Metallophytes include zinc violet (*Viola calaminaria*), plantain (*Plantago lanceolata*), alpine pennycress (*Thlaspi caerulescens*), *Cochlearia* spp, common bent (*Agrostis capillaris*), spring sandwort (*Minuartia verna*), sea thrift (*Armeria maritima*)^117^ and many others. Many members of the Brassicaceae family (90 species, about ¼ of the family) tend to hyperaccumulate metals from the soil.

Industrial hemp (*Cannabis sativa*) has a great potential for metal hyperaccumulation, which unfortunately has not been utilised for legal reasons.^118^ However, hemp grown on metal-contaminated soils offers a wide range of potential biomass uses,^118^ with the necessary further testing (*e.g.* fibre strength, chemical composition) to mitigate the restrictions on the use of *Cannabis sativa* biomass resulting from remediation practices, but also to ensure compliance with food and safety guidelines.^119^

In recent years, significant progress has been made in elucidating the mechanisms responsible for metal accumulation and detoxification in plants, including chemical and microbiological components, and in optimising field management practices to maximise the remediation potential by hyperaccumulation^120,121^ reported hyperaccumulation of metals in plant tissues, with concentrations reaching up to 3% by weight, without exhibiting phytotoxic symptoms. More than 500 plant species have been identified as hyperaccumulators of metals and metalloids,^122^ which represents approximately 0.006% of all angiosperms. Notably, about 75% of these hyperaccumulators have the ability to hyperaccumulate Ni.^123^ The Brassicaceae family is the richest in hyperaccumulators, though hyperaccumulators are found in over 34 different plant families. Within Brassicaceae, both Zn and Cd hyperaccumulators are abundant, particularly in the genus *Noccaea* (*e.g. N. caerulescens*, formerly *Thlaspi caerulescens*). Some *Noccaea* species/populations hyperaccumulate Ni in their natural serpentine soil environments and are capable of hyperaccumulating Zn under controlled conditions. Interestingly, hyperaccumulators are restricted to the genera *Noccaea* (Zn and Cd) and *Odontarrhena* (Ni), suggesting a monophyletic origin of hyperaccumulation. The *Alyssum* species that hyperaccumulate Ni do not accumulate Zn.^123^ These examples raise important questions about the evolution of hyperaccumulation mechanisms in plants.

During the last three decades, various selection factors were hypothesized to have lead to evolution of hyperaccumulation,^124^ such as: drought tolerance, allelopathy, increased metal tolerance, *etc.* Among the populations of hyperaccumulating species, due to a large variability in the capacity to tolerate and accumulate metals, there was an opportunity to analyse the underlying genetic determinants of the different variations, which opened a possibility of specifically selecting/breeding/genetically engineering a hyperaccumulating ideotype suitable for the remediation of specific metal-contaminated soils. A systematic study of metallophytes can identify priority candidates for the remediation of metal-contaminated soils and the development of environmentally friendly approaches based on the removal of contaminating metals from the soil (Fig. 1).

### 8.2. Bioashes and metal-contaminated soils

**Fig. 5:**
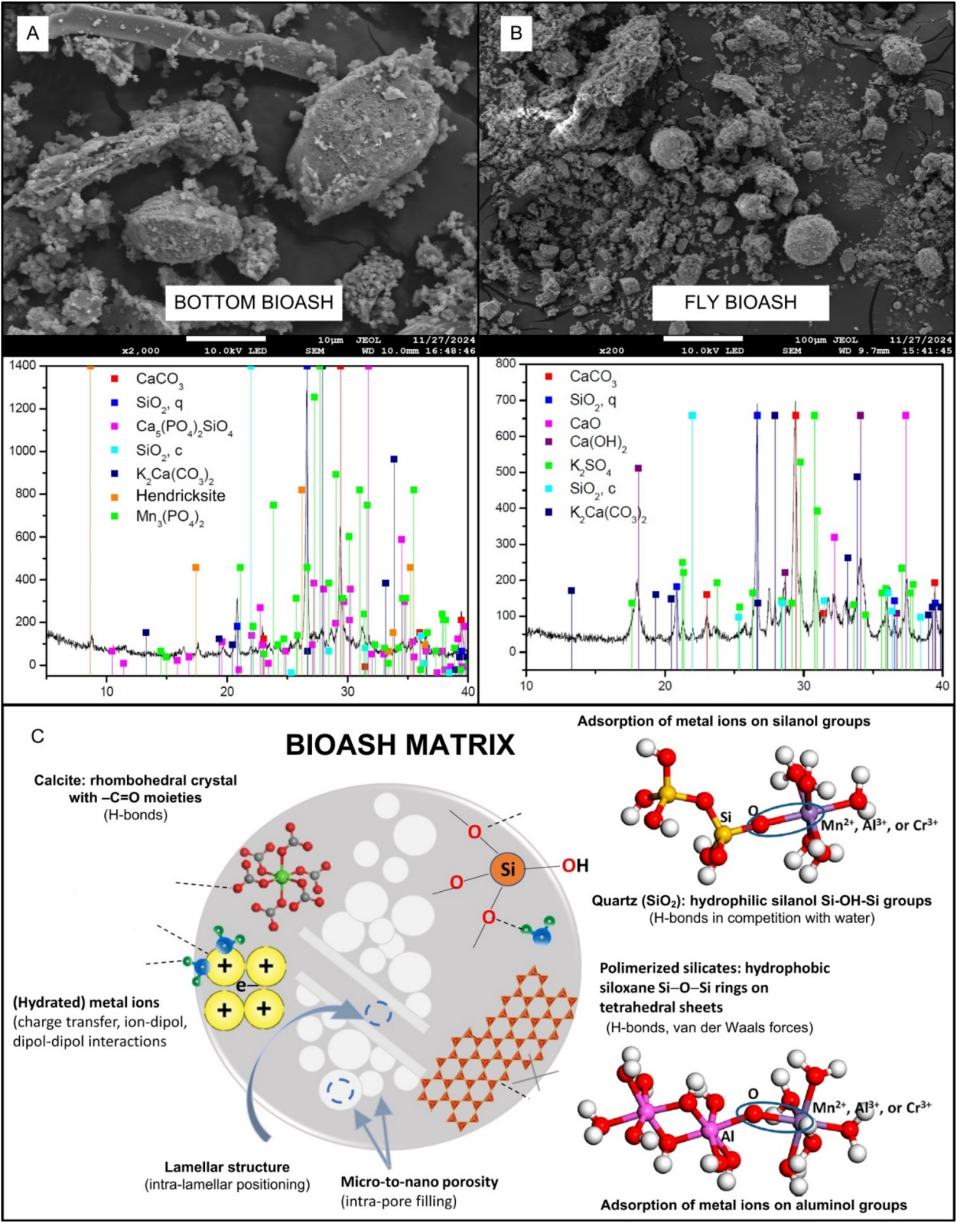
Characterization of bottom (A) and fly (B) bioash by scanning electron microscope and X-ray diffraction, with schematic representation of the most relevant biogeochemical reactions of metals in the presence of bioash matrix (C) (reproduced (adapted) with permission from ref. 100 Copyright© 2024, Elsevier and ref. 125 Copyright© 2019, American Chemical Society).

The oxidation of various biological residues (from forestry, agriculture or household) in different plant facilities (for electrical or thermal energy production, dryers, *etc.*) produces bioash, a reactive inorganic material characterised by an extremely complex composition and alkalinity (pH > 12), a variety of minerals, phytonutrients and trace elements^13^ (Fig. 5). In contrast to biochar (see the next section), with which it shares the same origin, the carbon content in bioash is low (<1% w/w), but has different ecological effects, is more alkaline due to the specific production conditions and has unique physicochemical properties (Fig. 5). For instance, principal component analysis (PCA) of 37 different types of bioashes explained 71% of the variation in their composition. The PC1 explained more than 41% of the variation, with high concentrations of pozzolanic oxides (Si, Al, Ti, Fe) being most influential. The PC2 explained almost 20% of the variation, predominantly with alkali oxides (Na, K), and PC3 explained almost 10% of the variation, predominantly with MgO.^126^ One of the key roles of bottom ash type used as soil amendment is in controlling soil biochemistry, due to high Si content in ash matrix, leading to increased metal adsorption capacity and shortening the time to reach equilibrium (ref. 127 and Fig. 5).

According to the studies of,^128^ the mobility of metals and their uptake by the corresponding biota is influenced by the addition of alkaline bioash to clay soils, which would change the biogeochemistry of metals in the soil. For example, in soils contaminated with metals, the addition of fly ash (5% w/w) reduced the leaching of Cu and Pb by >91% and >87%, respectively, due to the increased number of chemisorption sites and altered soil pH from 4.1 to 6.8; hence, the uptake of both metals by plants and bacteria in the soil decreased.^129^ The combined application of fly ash from wood and coal with peat was also more effective in terms of chemisorption of Cu and Pb than their separate application. Fly ash was effective in removing various metals from aqueous solutions (Fe > Cu > Zn > Mn)^130^ due to an increase in the pH of the liquid (from 4.2 to 8.0), which shifted the biogeochemistry of the metals towards physical adsorption at the interfaces of the ash and/or chemical deposition.^5^

Recently, two types of bioashes, rice husk ash (RHA) and sugarcane bagasse ash (SBA), were approved as soil amendments to immobilize metals in contaminated soils, reducing the metal toxicity and health risk associated with metals in wheat.^131^ Specifically, SBA proved more effective, reducing Cr, Ni, Cu, Zn, and Cd in seeds by 13.5%, 33.8%, 17.6%, 7.8%, and 10.0%, respectively, compared to RHA reductions of 6.8%, 16.9%, 8.8%, 3.9%, and 5.0%, with metals accumulating most in roots and least in seeds. Absorption and dissolution processes could explain some of results of the aforementioned studies under strong pH influence. This is because the proportion of cationic metal forms increases at low pH values, whereas anionic forms dominate at high pH values. The addition of bioash to soil can significantly alter key physico-chemical pedovariables, influencing metal biogeochemistry, including shifts in soil pH, electrical conductivity, bulk density, and water-holding capacity,^100^ along with increases in carbon and phytonutrient content.^131^ These modifications underscore the potential of bioash to impact soil functionality and nutrient cycling, making it a valuable tool for soil management in contaminated or degraded ecosystems.

### 8.3. Biochair and metal-contaminated soils

**Fig. 6:**
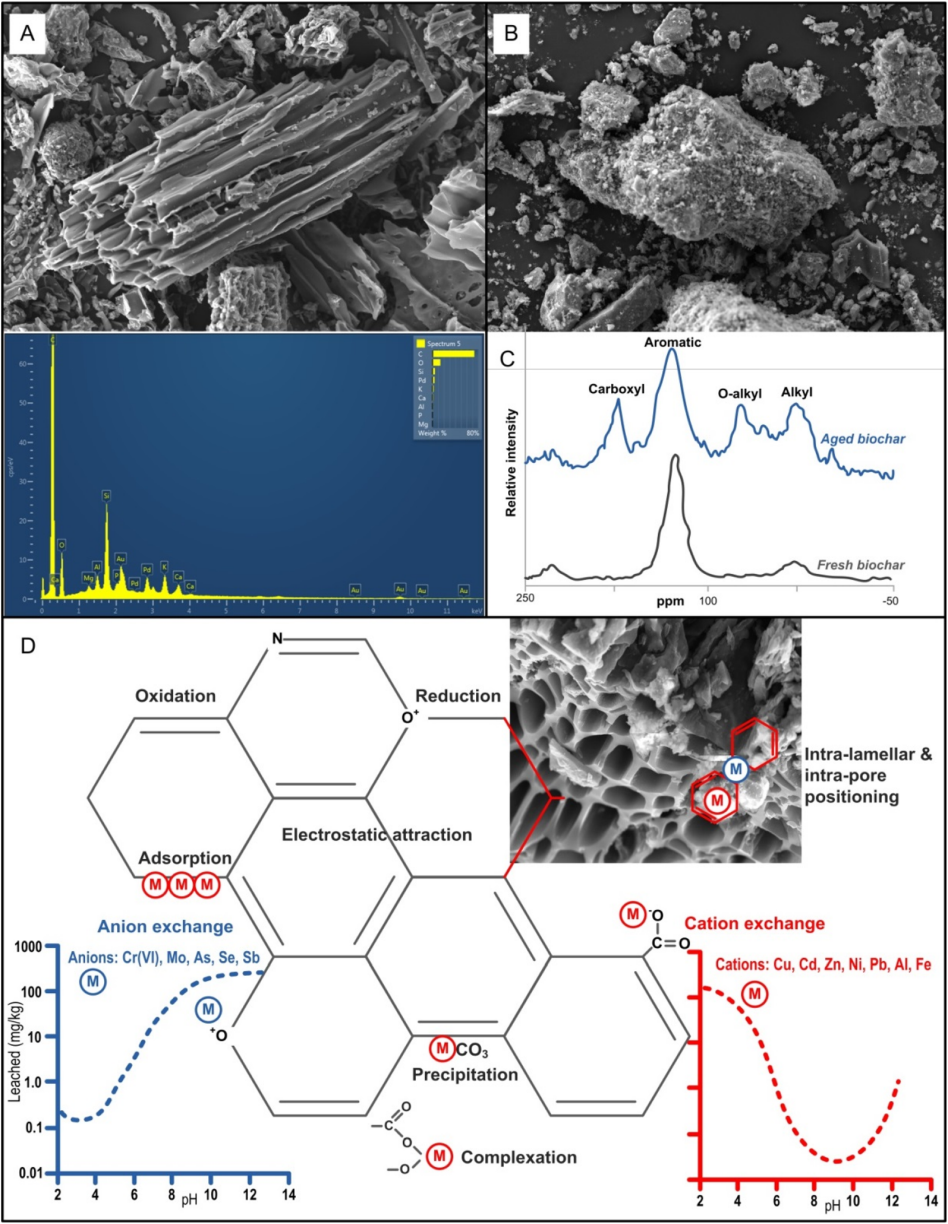
Characterization of wood chips-derived biochar by scanning electron microscope (SEM) and energy-dispersive X-ray spectroscopy (A) and of sewage sludge-derived biochar by SEM (B),^62^ with solid-state ^13^C CP-MAS NMR (Cross-Polarization Magic Angle Spinning Nuclear Magnetic Resonance) spectra of fresh and aged biochar (reproduced (adapted) with permission from ref. 133, Copyright© 2015 Taylor & Francis), and schematic representation of the most relevant biogeochemical reactions and immobilisation mechanisms of metals/metalloids in the presence of biochar matrix and different pH conditions (D) (reproduced (adapted) with permission from ref. 134 Copyright© 2021, Elsevier).

Biochar is an organic material produced by pyrolysis in which the temperature inside the container is gradually raised to 300–700 °C (Fig. 3), using filters to capture and store potential pollutants as well as C.^132^ Biochar is C rich (containing >50% w/w C), and one of the most efficient matrices to convert C into a stable form that can then be incorporated into the soil as a soil conditioner.^118^ Consequently, as a very stable and porous substance, biochar has gained significant attention due to its potential benefits in reducing the availability and leaching of metals (Fig. 6), and ultimately limiting the accumulation of metals in edible tissues.^135^ For example, the addition of biochar at 5% w/w reduced the availability of Pb, Cd and Zn by around 50%.^136^ In the study by ref. 137 biochar derived from rice straw and bamboo significantly reduced the extractable fraction of metals in the soil as well as the fractions of organic complexes, CaCl_2_-and DTPA. The capacity of biochar to stabilise metals in the soil has been demonstrated in many studies in the field and under controlled conditions but stabilization has not been considered effective enough to be used as a practical solution to the problem of soil metal contamination. To achieve the desired level of toxic metal stabilisation, options include expanding the range of feedstocks, optimizing the pyrolysis conditions used to produce biochar, and increasing the rate of biochar application.^138^ However, due to the costs associated with biochar production and application, the maximum biochar application rate tested at 20% w/w is still too high to be of practical use.^72^ Other soil remediation approaches, such as phytoremediation,^139^ myco-remediation, or the removal of contaminated soil, may incur costs similar to or higher than biochar production and application, providing alternative strategies for addressing metal contamination.

The widespread use of biochar in agroecosystems is hindered by the fact that high biochar application rates are not practical for farmers or environmental engineers^140^ because the application of high amounts of biochar or bioash would increase soil alkalinity/salinity, thus jeopardising plant development and growth.^141^ Extensive research is currently being conducted to identify and characterise an alternative method that can improve the efficiency of biochar in metal stabilisation at relatively low application rates, thus achieving the remediation of metal-contaminated soils at an acceptable price and without negative effects on plant growth.^120^ To improve the stabilisation of metals in soil, many studies have focused on biochar modification^142^ by using organic solvents and acids, iron compounds and hydroxides.^143^ Potentially viable options are *e.g.* increasing cation exchange capacity by increasing the number of functional groups on the surface of biochar, which can significantly improve its effectiveness and would obviate a need for high application rates of biochar to the soil.^144^ Optimising the specific surface area of biochar is also crucial. By increasing the specific surface area, the sorption capacity for metals can be significantly improved, which would further increase the efficiency of biochar in removing heavy metals from the environment.^145^ Biochar can be modified by adding other materials or chemicals that improve its adsorption properties. For example, impregnating biochar with metal oxides or nanoparticles can increase its capacity to bind pollutants. By combining these approaches, it is possible to develop biochar with several enhanced properties to underpin greater efficiency in remediation of soil and water while requiring lower rates of biochar. These advanced solutions would not only reduce costs and environmental impact, but also contribute to more sustainable resource management and environmental protection.

So far, most of the biochar modifications have relied on using one compound or solution (*e.g.* Fe compounds, HNO_3_, H_2_SO_4_), with very few studies testing multiple modifiers simultaneously.^146^ Hence, a potential of using multiple additives to improve biochar properties, and/or applying biochar with multiple modifiers to improve a range of specific soil properties^147^ is yet to be explored fully.

Due to the complexity of different soils combined with the limitations of currently available analytical techniques, it is still difficult to accurately determine the mechanisms by which biochar immobilise/removes metal(loid)s.^120^ Several relevant mechanisms have been proposed, such as oxidation [*e.g.* Hg^0^,^148^ reduction (*e.g.* Se^6+^),^149^ intra-lamellar and/or intra-pore positioning (*e.g.* Zn^2+^, Cu^2+^),^150^ physical adsorption, ion exchange (*e.g.* Cr^3+^, Cd^2+^),^151,152^ co-precipitation (*e.g.* Pb^2+^),^153^ electrostatic adsorption and surface complexation (*e.g.* Zn^2+^, Cd^2+^, Cu^2+^; As^3+^, As^5+^) with functional groups containing oxygen and an electron located in a pi shell or a double or triple bond, or in some cases in a conjugated pi shell^134,154^ (Fig. 6).

Dominance of a specific metal-immobilising mechanism and its efficiency are strongly dependent on type and aging of particular biochar,^155^ pH conditions (*e.g.* the ratio of anionic and cationic metal forms), SOM and metals properties (*e.g.* ionic radius, valence)^156,157^ (Fig. 6). For instance, recently performed meta-analyses revealed that soil pH exerted the greatest influence on metal bioavailability in soils amended with biochar, with soil texture, aging time, biochar pyrolysis temperature, metal species and applied dosage following in significance.^155^ In soil amended with biochar under alkaline pH conditions, enhanced metal immobilisation can be explained by (i) deprotonation of acidic radicals and their preference for anionic metal forms, (ii) the release of Na^+^, K^+^, Cl^−^, various phosphate forms and OH^−^, promoting the formation of relatively stable inner-sphere metallo-complexes and/or (iii) metal precipitates (with CO_3_^2−^, H_2_PO_4_^−^, HPO_4_^2−^ or OH^−^)^155^ (Fig. 4).

The oxidation of biochar surfaces, expected to produce negatively charged organic functional groups, plays a significant role in determining CEC and pH of biochar, consequently impacting the leaching patterns of metals (Fig. 4). Moreover, compared to fresh biochar, aged biochar contains a higher concentration of carboxyl, aromatic, *O*-alkyl, and alkyl surface functional radicals, thereby enhancing its capacity for retaining metals^133^ and reducing bioavailability of metals in soils amended with biochar^155^ (Fig. 6).

Some studies on adding biochar to soils reported only a slight change in soil pH and cation exchange capacity, possibly related to the removal of alkaline components from the tested biochar during the modification process.^158^ It can be inferred that ion exchange and co-precipitation of metals (controlled by alkaline groups and pH) may not be the main mechanisms for enhanced stabilisation of metals in contaminated soils.^159^ Increasing the number of functional groups (–SH, –OH, –COOH) in biochar matrix (Fig. 6) has been shown to enhance its remediation potential by significantly increasing its effective surface area and total pore volume, making it an important approach for biochar modification.^160^ Higher efficiency in metal stabilisation was achieved by modified (compared to unmodified) biochar,^161^ suggesting perhaps the most important mechanism for Cd stabilisation and remediation of Cd, Pb and Zn pollution in various soils by effectively reducing availability and leaching,^129^ through surface complexation with functional groups.^72^ The harsh conditions in metal-polluted soils are at least partially improved by converting metals into more stable fractions.^162^ In addition to physico-chemical changes in metal binding and speciation (Fig. 4), biochar also improves a range of other pedovariables, including enhanced soil microbiomes and enzyme activity,^163^ water retention, nutrient availability,^118,156^ and many others (see ref. 164).

## 9. Estimation and prediction of metal dynamics in soils using artificial intelligence

Artificial intelligence (AI) is a branch of computer science focused on developing algorithms that replicate human brain functions, including the ability to learn from specific patterns in datasets. Machine learning (ML), a subset of AI, enables computers to learn without explicit programming by using statistical methods that allow machines to improve through iterations.^165^ Unlike traditional statistical models, ML algorithms are highly flexible as they do not require assumptions about the data distribution. Instead, they learn from the patterns in the training datasets. Among the most widely used and adaptable ML models is the artificial neural network (ANN) that effectively handles varying levels of linearity and complexity in datasets.^166^ Other popular ML models include support vector machines (SVM), random forests (RF), fuzzy techniques, and k-nearest neighbours (KNN) (Table 3). Given the complex, nonlinear, and chaotic nature of metal–soil–plant interactions, ML models often yield more accurate predictions compared to classical models. However, there is no one-size-fits-all model. Developing an ML model requires selecting the optimal combination of hyperparameters, which significantly influences model accuracy. Although limited literature exists on using ML algorithms for metal identification and prediction, there have been substantial advancements in simulating metal behaviour in soils over the past two decades.

**Table 3:** Performance of machine learning (ML) models in metal analysis and prediction^a^

Description	Best model	Other models tested	Reference
Estimation of Cd and Pb in polluted soil, Gilan province, Iran	ANN	ANFIS	167
Estimation of Fe, Mn, and Zn in Mount Ida, Turkey	ANN		168
Estimation of Ni, Pb, Cr, Hg, Cd, As, Cu, and Zn in polluted soil, Huanghua City, China	Hybrid LASSO-GA-BPNN	SVR, RF	169
		RF, ordinary kriging	
Estimation of Zn, Cu, Cr, and Pb in topsoil of the Dammam area, Saudi Arabia	ANN and SVR		170
Prediction of Pb and Cd in soils from mining areas	BPNN		171
Prediction of Pb from sediments of two bays in Queensland, Australia	XGBoost	ANN, SVM, RF	172
Analysis and calibration of trace elements (Pb, Cu, Ni, Zn, co, Cd, As, Sc, Hg, Mn, Cr, Ti, Sb, Sr, V, Ba) in soil of a waste disposal site, Rajbandh, Khulna, Bangladesh	ANFIS	ANN, SVM	173
Multivariate calibration model prediction of Ag concentrations in different soils, Lyon, France	ANN	BPNN	174
Prediction of Cr concentration in subarctic soil, Novy Urengoy, Russia	MLP	GRNN	175
Prediction of Cd, As and Pb in soil of mining area, Jiangsu Province, China	RF	SVM, ELM, PLS	176
Prediction of immobilisation of metals (Cu, Zn, Pb, Cd, Fe, Ni and Mn) in biochar-amended soils from 15 different studies	RF	ANN, SVM	157
Identification of the source and spatial prediction of metals (Zn, Pb, Hg, Ni, Cu, Cr, Cd) and As in peri-urban soil, Hefei City, China	SVM and RF	SVM, ANN	177

a ANFIS – adaptive neuro-fuzzy inference system; ANN – artificial neural network; BPNN – back-propagation neural network; ELM – extreme learning machine; GA – genetic algorithm; IDW – inverse distance weighting; LASSO – least absolute shrinkage and selection operator; MARS – multivariate adaptive regression spline; PLS – partial least squares; RF – random forest; SVM – support vector machine; SVR – support vector regression; XGBoost – extreme gradient boosting.

Single- or multi-stage digestion/extraction using a wide range of elemental and isotopic analytical tools is typically needed to determine metal forms and dynamics in soils and the related (microbial, plant) matrices,^2^ which requires time, resources and labour. Artificial intelligence techniques are proving to be important alternatives. They may be superior to standard chemical analyses for solving spatial and temporal dynamics of metals in soil. Furthermore, AI approaches can predict the efficiency of metal immobilization in wide range of scenarios, identifying optimal environmental conditions and a selection of amendment(s) considering their complex and reactive matrices (*e.g.* biochar *vs.* bioash) to maximize metal immobilization (Fig. 4). In addition, the use of AI can (i) explain how the prevailing atmospheric conditions can affect the dispersion of metals from source locations, (ii) suggest crops that may be suitable for cultivation in specific metal-contaminated soils (Fig. 1), (iii) take into account various spatio-temporal hydro-geo-pedogenic processes to predict the range of metal concentration in a given area (Table 3). Recently,^157^ have successfully applied three AI techniques to predict metal immobilization in biochar-amended soils, revealing that N content in the biochar (ranging from 0.3% to 25.9%) and its application rate (ranging from 0.5% to 10%) as the most influential factors, with the causal analysis indicating the following hierarchy of empirical categories for metal immobilization efficiency: biochar properties > experimental conditions > soil properties > metal properties. In addition,^168^ developed the ANN models to determine the dynamics of metals such as Fe, Mn and Zn as influenced by the concentrations of Ca, K and Mg in soil samples obtained from different altitudes. Recently,^172^ proposed and validated algorithms against different AI models (ANN, SVM, RF) for Pb prediction by using 13 input variables (*i.e.* total metal concentrations) in the sediments of two Australian bays, with Zn being the most effective predictor for Pb, followed by Ni and Cu.

The tuning of the hyperparameters in the AI predictive models can be achieved by an advanced AI optimization^172^ to make modelling performance more reliable and applicative, which is especially valid under conditions of limited technical and logistical resources, *e.g.* in developing countries. Namely, one of the main advantages of AI algorithms is a possibility of using different big data repositories, which in combination with an appropriate technical support (*e.g.* supercomputers) enables fast processing of numerus iterations and generation of relevant scenarios.

Among the various AI models used to predict metals in soils, ANN is the predominant one; however, some newly introduced hybrid machine learning models have been confirmed as superior for metals in specific pedospheres.^178^ Consequently, derived AI approaches could assist and significantly accelerate metal detection in the soil as well assessing economic, health and environmental impacts in various conditions, thus improv policies and regulations to diminish metal contamination and secure the sites that may be sources of metals.

## 10 Conclusions and future perspectives

For restoration of metal-contaminated soils there are initial requirements to characterize the contamination type and source, as well as the spatial range and depth of soil contamination.^179^ Contemporary legislation designed for the protection of public and environmental health is based purely on the chemical characterization within each tested site. Particular attention should be focused on potentially toxic metals that may enter the food chain, water bodies or atmosphere.^7^ Given that soil characterization for legislative and regulatory purposes is done based on total metal concentrations, a substantial improvement to underpin successful remediation strategies is needed *via* determining metal speciation that governs bioavailability and mobility.^180^ As a risk-minimization strategy, decision makers seek solutions that can guarantee the removal or *in situ* immobilisation of the metal contaminants in the most cost-effective manner while preserving both public and ecosystem health.^181^ The impact of military activities, including the manufacture and disposal of weapons, the utilization of ammunition during military exercises, and engagement in conflicts and wars, is frequently underestimated despite their significant and multiple contributions to metal pollution (Fig. 1). These activities include the releases high loads of toxic metals as common constituents of ammunition,^182^ coatings, electronic devices, *etc.* The shells and bullets used in firing ranges can remain in the soil for long periods, resulting in persistent metal pollution.^183^ A recent study by^184^ found that military activities have led to the accumulation of Pb, Cd and Hg in soils around military bases. Similarly, a study by^185^ found that military activities in Malaysia have resulted in elevated levels of Pb and Cu in soils around firing ranges. The Russian–Ukrainian war is expected to have a significant and long-lasting multiplicative impact on the levels of metal contamination in various environmental niches, not only in the affected regions, then globally.^186^ A massive destruction of infrastructure has already resulted in the release of metals and other pollutants into the environment, hindering efforts to achieve a metal-clean environment, as well as energy and food security.^186^ The most recent studies confirm significant increases in metal concentrations (Pb, Cd, Hg, Cu, Zn, Ni, Co, Sn, Mn, Se, Al) in the soils of Ukrainian regions affected by the Russian invasion.^187,188^ This is particularly significant, as Ukraine's chernozems are among the most productive and high-quality soils on Earth, and their restoration and remediation after the war will be crucial.

Due to a greater awareness based on the scientific knowledge, governments are dealing with contaminated soils and their implications for human and ecosystem health, by funding the further development of remediation strategies while simultaneously assessing, mapping and classifying metal contamination within their borders. However, increasing environmental pressures due to various anthropogenic activities (*e.g.* plastic, persistent organics) exacerbate the problems with metal-contaminated soils due to synergistic negative repercussions on environmental health, food safety and security. For example, recent studies warn that addition of micro-plastic^120^ or combined application of humates and chloride salts^9^ to Cd-contaminated rhizosphere markedly change biochemical reactions, metabolites and their pathways, increasing Cd availability and promoting its uptake by crops. Importantly, the interactions among various pedovariables lead to multicollinearity, making specific and synergistic effects extremely complex to discern. However, due to possibility of fast processing of numerous predictors under different relevant scenarios, the fast-developing algorithms driven by AI appear to be a promising approach to optimising the management of metal-contaminated areas, addressing the numerous knowledge gaps in metal interactions.

## Consent for publication

All authors agreed to publication.

## Data availability

All data will be made available upon request.

## Author contributions

GO conceptualisation, funding and project administration. GO, JS and JH graphical data presentation. GO, JS, SR, RD, MIR, MB, MSS, JH and ZR drafted the manuscript. ZR discussed, revised and amended the text. All authors finalised the review.

## Conflicts of interest

The authors declare no competing interest.
